# GeoSlide-XMamba: A Spectral-Topographic Boundary-Aware State-Space Network for Landslide Semantic Segmentation

**DOI:** 10.3390/s26134146

**Published:** 2026-07-01

**Authors:** Yi Tang, Fei Zhao, Guojian Feng, Hongwen Yang, Luhao Gao, Lin Zheng, Weixia Zhou

**Affiliations:** 1 College of Architecture and Civil Engineering, Kunming University, Kunming 650214, China; ytangyn@kmu.edu.cn (Y.T.); fengguojian2000@kmu.edu.cn (G.F.); yanghw1974@kmu.edu.cn (H.Y.); zwx13700607481@kmu.edu.cn (W.Z.); 2 School of Earth Sciences, Yunnan University, Kunming 650500, China; 3 Yunnan Surveying and Mapping Geographic Information Technology Development Co., Ltd., Kunming 650106, China; gaoluhao001@outlook.com; 4 College of Smart Agricultural Engineering, Yunnan Vocational and Technical College of Agriculture, Kunming 650031, China; lzhengyn@163.com

**Keywords:** landslide mapping, semantic segmentation, Landslide4Sense, Sentinel-2, DEM, mamba, state-space model, boundary-aware learning, Jinsha River Basin

## Abstract

**Highlights:**

**What are the main findings?**

- GeoSlide-XMamba integrates dual-branch spectral-topographic encoding, STAF++ fusion, terrain-conditioned selective state-space scanning, and boundary-aware refinement for landslide semantic segmentation. On the Landslide4Sense validation split, the model achieves the best overall performance among the compared methods, with F1 = 0.673, IoU = 0.507, Boundary-F1 = 0.466, and HD95 = 3.45 pixels. Multi-seed experiments further show stable improvements over the direct 14-channel concatenation baseline, while expanded Jinsha River transfer inference produces spatially coherent probability responses in complex mountain terrain.
- The expanded evaluation includes HD95, mean ± standard deviation, 95% confidence intervals, boundary error diagnostics, a 4 × 4 Jinsha River transfer panel, misclassification analysis, and terrain-conditioned direction-weight inspection. Across five seeds, GeoSlide-XMamba obtains F1 = 0.673 ± 0.003 with a 95% CI of [0.670, 0.676], indicating that the observed improvement is not limited to a single favorable random initialization.

**What are the implications of the main findings?**

- These findings suggest that terrain information is most useful when it is treated as a geomorphic constraint that shapes feature propagation rather than as ordinary input channels concatenated with spectral bands. The terrain-conditioned scan gate enables DEM- and slope-derived morphology to regulate long-range dependency modeling, which improves boundary coherence and reduces confusion between landslides and spectrally similar exposed surfaces. This design may also support future multi-source extensions that incorporate SAR backscatter, InSAR deformation fields, or event-based change features for geohazard monitoring.

**Abstract:**

Rapid and reliable landslide mapping from satellite observations is essential for hazard assessment, emergency response, and reservoir-area risk management, yet automatic segmentation remains challenging in mountainous regions because landslide scars are spectrally heterogeneous, terrain-constrained, morphologically irregular, and frequently confused with other exposed surfaces. This study proposes GeoSlide-XMamba, a terrain-conditioned spectral-topographic boundary-aware state-space network for pixel-wise landslide semantic segmentation. The model first separates Sentinel-2 spectral bands and DEM/slope-derived topographic layers into modality-specific branches, integrates them through spectral-topographic adaptive fusion (STAF++), and then performs terrain-conditioned selective state-space scanning in the XMamba bottleneck. Unlike direct token concatenation, the proposed bottleneck uses terrain descriptors to dynamically weight directional selective scan branches so that long-range feature propagation is guided by slope-related morphology. Boundary-aware decoding, signed-distance supervision, and hard-negative mining are further introduced to improve inventory-oriented geometric quality and suppress common false positives. Experiments were conducted on the Landslide4Sense benchmark using 14-channel multispectral-topographic inputs. Among the compared methods, GeoSlide-XMamba achieved the highest validation performance under a unified five-seed protocol, with precision = 0.729, recall = 0.626, F1-score = 0.673, IoU = 0.507, kappa = 0.666, Boundary-F1 = 0.466, and HD95 = 3.45 pixels. Five-seed experiments produced F1 = 0.673 ± 0.003, IoU = 0.507 ± 0.002, Boundary-F1 = 0.466 ± 0.002, and HD95 = 3.45 ± 0.13 pixels, with a 95% CI of [0.670, 0.676] for F1. Relative to the strong 14-channel concatenation baseline, the proposed model improves mean F1 by 0.045 and reduces HD95 by 1.42 pixels. Expanded qualitative inference on Jinsha River patches indicates that the learned spectral-topographic representation transfers plausibly to high-relief reservoir-canyon terrain. These results show that terrain-conditioned state-space modeling can improve both segmentation accuracy and boundary geometry for remote sensing landslide mapping.

## 1. Introduction

Landslides pose persistent threats to hydropower infrastructure across mountainous southwestern China. Along the Jinsha River, deeply incised valleys host cascading reservoir systems, and impoundment-induced water-level cycling, pore-pressure changes, and toe erosion keep many slopes at the edge of stability for years [1,2,3,4]. Failures in these settings rarely remain localized: impulse waves, barrier lakes, and downstream debris flows can propagate far beyond the initial rupture zone [5,6]. The Jinsha River Basin exemplifies this pattern—its combination of high relief, active tectonics, and dense hydropower development has produced repeated landslide disaster chains [7,8,9,10,11]. The scale of these risks became concrete in 2018 at Baige, where two successive slope collapses deposited roughly 24 × 10^6^ m^3^ and 9 × 10^6^ m^3^ of debris into the river, damming the channel twice within a month [8]. The resulting barrier-lake outburst floods caused direct economic losses of approximately 6.8 billion RMB (≈US $0.96 billion) [12]. Events of this kind leave little doubt that reservoir-canyon corridors need faster and more reliable landslide mapping.

Inventory-quality mapping underpins all downstream hazard and risk analysis [13], and its reliability sets an upper bound on whatever assessment follows [14]. In canyon settings, however, optical remote sensing faces compounding difficulties. Terrain shadow is pervasive, vegetation phenology shifts between seasons, and landslide scars share spectral signatures with river bars, quarries, and freshly plowed terraces [15,16]. Narrow valley geometry further degrades class separability by concentrating diverse land-cover types within small spatial extents. Under these conditions, spectral information alone is insufficient; geomorphic context—slope gradient, local relief, and drainage position—must also inform the mapping process [17,18].

Deep learning has been applied to satellite-based landslide detection with increasing frequency over the past decade. High-resolution UAV studies demonstrated early successes [19], and instance-segmentation architectures such as Mask R-CNN were subsequently adapted for landslide object extraction [20], though results degraded where terrain shadow was severe. SAR-based methods offered weather-independent coverage [21] at the expense of layover and foreshortening in steep terrain. Transfer learning eased data scarcity [22], and the Landslide4Sense benchmark [23,24,25] established a shared evaluation protocol that underlined the value of including DEM and slope channels alongside optical bands. Even so, the dominant fusion strategy remains channel-wise concatenation, which treats terrain layers as additional spectral bands and ignores their distinct physical meaning [26]. Recent work has started to address segmentation quality more directly: Li et al. [27] introduced a two-phase detect-then-segment pipeline that improved object-level completeness, while Li et al. [28] showed that combining STAPLE consensus masks with fully connected CRFs sharpened loess-landslide boundaries. Richer training resources have also become available through the CAS Landslide Dataset and Sen12Landslides [29,30], broadening the geographic and sensor diversity of labeled data for multi-source landslide mapping.

Architecturally, convolutional encoders are constrained by local receptive fields and struggle to capture elongated landslide scars that may stretch hundreds of meters along a slope [31]. Transformer-based designs extend the modeling range through global self-attention, but the quadratic token-count cost limits their practical use on large or densely sampled inputs [32]. Mamba-family state-space models—Vision Mamba, VMamba, U-Mamba, VM-UNet, UNetMamba, and PyramidMamba—occupy a useful niche between the two, propagating contextual information across long spatial extents at linear complexity [33,34,35,36,37,38]. Yet, to date, no state-space architecture has been applied to landslide segmentation, and none conditions its scanning directions on geomorphic structure: propagation still follows fixed image-grid axes regardless of how terrain is oriented. A related limitation is that most segmentation pipelines optimize pixel overlap while leaving boundary geometry largely uncontrolled; F1 and IoU can look acceptable even when predicted outlines are too fragmented or too smooth for polygon-based inventory workflows [39].

A practical question follows: can the spatial propagation mechanism inside a segmentation network be conditioned on terrain morphology, and does doing so yield landslide masks whose boundaries are accurate enough for direct vectorization? The question matters because current inventory practice still relies heavily on manual or semi-automatic digitization precisely where automatic methods produce geometrically poor outlines. If a model could infer that a scar is likely to extend downslope along a convex break rather than across contour lines, both false-positive confusion with spectrally similar river bars and boundary fragmentation along shadow edges should decrease. We expect that replacing grid-fixed state-space scanning with terrain-conditioned directional scanning and coupling it with explicit boundary supervision will improve not only overlap-based metrics but also the geometric fidelity required for operational landslide inventories in reservoir-canyon terrain.

This study introduces GeoSlide-XMamba along these lines. Spectral and topographic inputs enter the network through separate encoder branches so that reflectance and terrain variables retain their distinct representations until an explicit fusion step. Spectral-topographic adaptive fusion (STAF++) then combines the two streams through a learned gate that modulates the relative contribution at each spatial location. The fused features are passed to a terrain-conditioned XMamba bottleneck, where DEM- and slope-derived descriptors predict direction-specific weights for multiple selective scan branches; long-range dependencies are thereby aggregated preferentially along terrain-consistent directions rather than along image-grid axes. On the decoder side, boundary-aware refinement, signed-distance supervision, and hard-negative mining target the geometric quality of predicted masks. Experiments use the Landslide4Sense benchmark [40] with 14-channel input—twelve Sentinel-2 multispectral bands [41], one ALOS PALSAR DEM layer, and one slope layer [42].

Compared with existing landslide segmentation architectures, the dual-branch design with STAF++ is the first to preserve modality-specific encoding all the way to an explicit cross-source fusion gate rather than concatenating channels at the input. The terrain-conditioned bottleneck reformulates selective scanning so that geomorphic context—not image-grid proximity—governs directional feature propagation, which is a departure from existing Mamba variants that treat all scanning directions equally. The addition of signed-distance supervision, HD95 evaluation, and systematic hard-negative analysis provides a more inventory-oriented assessment of geometric quality than the pixel overlap metrics used in most prior work. Under this design, GeoSlide-XMamba reaches five-seed mean F1 = 0.673, IoU = 0.507, Boundary-F1 = 0.466, and HD95 = 3.45 pixels, with F1 = 0.673 ± 0.003. Section 2 describes the dataset, study area, network architecture, loss functions, and experimental protocol. Section 3 presents the main comparison, ablation experiments, input-modality analysis, and qualitative Jinsha River transfer results. Section 4 discusses the findings. Section 5 concludes.

## 2. Materials and Methods

### 2.1. Landslide4Sense Benchmark Dataset

The Landslide4Sense benchmark [40] provides 128 × 128 image patches with 14 channels, including 12 Sentinel-2 multispectral bands and two ALOS PALSAR-derived topographic layers, namely DEM and slope. The Sentinel-2 channels provide visible, red-edge, near-infrared, and shortwave-infrared information, which are useful for describing exposed soil, vegetation disturbance, moisture differences, and shadowed terrain [41]. The DEM and slope layers describe elevation structure and local terrain steepness [42]. This multi-source setting is suitable for landslide semantic segmentation because landslide scars may be spectrally similar to roads, river beaches, construction areas, agricultural terraces, or exposed bedrock, whereas topography provides additional information about where slope failures are physically plausible. Using this benchmark also allows all compared models to be evaluated with the same spatial patch size, input dimensionality, label definition, and validation protocol.

In the implementation used here, H5 image files and corresponding H5 annotation files were converted into a fixed directory structure. Each image was transformed into a 14-channel tensor, and each annotation was converted into a single-channel binary mask in which landslide pixels were assigned 1 and background pixels were assigned 0. The processed dataset contains 6342 training patches and 942 validation patches. Before training, each channel was normalized to reduce scale differences between optical reflectance and terrain variables. Normalization is particularly important because spectral channels and topographic layers have different physical units, value ranges, and spatial distributions. The main comparison and ablation experiments used a fixed training protocol, while a repeated-seed experiment was additionally conducted for the proposed model and the strong 14-channel concatenation baseline. The official Landslide4Sense test set labels are not publicly released, which precludes submission to the official leaderboard. The processed split used here is therefore designed as an internally consistent validation protocol for architectural comparison rather than a replacement for official benchmark evaluation.

**Figure 1:**
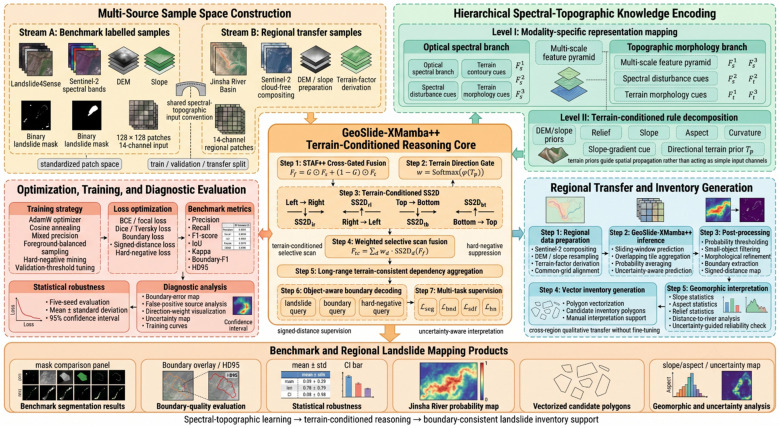
Overview of the GeoSlide-XMamba experimental workflow. The left panel presents the benchmark training and validation pipeline based on processed Landslide4Sense patches. The right panel illustrates the qualitative transfer inference procedure on 14-channel Jinsha River patches using the best checkpoint.

The Landslide4Sense benchmark was chosen as the primary evaluation dataset for several reasons. It is, to date, the only publicly available multi-source landslide segmentation dataset that pairs Sentinel-2 multispectral imagery with co-registered DEM and slope layers in a fixed 14-channel patch format, which directly matches the dual-branch input design of GeoSlide-XMamba. Alternative datasets such as the CAS Landslide Dataset [29] and Sen12Landslides [30] offer broader geographic coverage but use different channel configurations, spatial resolutions, or label definitions, and would therefore require non-trivial adaptation of both the input pipeline and the compared baselines. Evaluating on multiple benchmarks with different conventions in a single study risks conflating architectural effects with protocol-adaptation effects. A multi-dataset evaluation is planned as future work once a unified cross-benchmark protocol can be established (see Table 1 and Figure 1).

The Jinsha River transfer experiment is used as an additional cross-region qualitative check. Because no independent regional labels are used, this experiment is interpreted as transfer plausibility rather than as a quantitative accuracy assessment.

### 2.2. Jinsha River Basin for Qualitative Transfer Inference

The Jinsha River Basin was selected as a qualitative regional transfer area because it is one of the most landslide-prone mountain river systems in southwestern China. The basin is characterized by deeply incised valleys, steep relief, strong tectonic activity, active river erosion, and heterogeneous lithology. In this geomorphic setting, slope failures may be triggered or reactivated by rainfall, earthquakes, valley undercutting, reservoir-level fluctuation, road construction, and long-term gravitational deformation. Previous studies have reported active landslides, slow-moving deformation, and catastrophic failure chains along the Jinsha River using optical interpretation, InSAR monitoring, and geomorphic analysis [7,8,9,10,11]. These characteristics make the basin a meaningful target for examining whether a benchmark-trained model can produce plausible regional landslide probability outputs.

Benchmark validation and regional inference were treated separately in this study. Landslide4Sense provides standardized image patches with pixel-level labels, whereas the Jinsha River scenes contain more heterogeneous terrain and seasonal conditions and do not include independent regional ground truth. Under these conditions, transfer inference cannot provide quantitative accuracy evaluation, but it can still be used to examine whether the trained model produces spatially reasonable probability responses in a geomorphically different environment. The Jinsha River results are therefore presented only as qualitative transfer examples and are not interpreted as a completed regional landslide inventory.

For the transfer inference experiment, 14-channel patches covering parts of the Jinsha River Basin were prepared using the same channel configuration and normalization procedure as the Landslide4Sense training data. The best-performing GeoSlide-XMamba checkpoint was then applied directly to these patches without any fine-tuning. Probability maps were thresholded to generate candidate landslide masks, and boundary and signed-distance maps were derived from the masks for visualization purposes. Sixteen representative patches were selected to illustrate typical outputs across different terrain conditions. No quantitative accuracy assessment was performed at this stage, as independent regional labels were not available.

The transfer inference workflow is now described explicitly. Regional Sentinel-2 imagery is first composited within a fixed temporal window and resampled to match the Landslide4Sense patch convention. DEM-derived terrain layers are then generated using the same DEM/slope channel order and channel normalization strategy as in the benchmark data. The regional raster stack is divided into overlapping 128 × 128 patches, and the trained model is applied in a sliding-window manner without fine-tuning. Overlapping predictions are averaged to reduce boundary artifacts, after which probability thresholding, small-component filtering, boundary extraction, signed-distance transformation, and optional vectorization are performed. The resulting maps are interpreted as candidate probability outputs rather than as a validated regional inventory.

**Figure 2:**
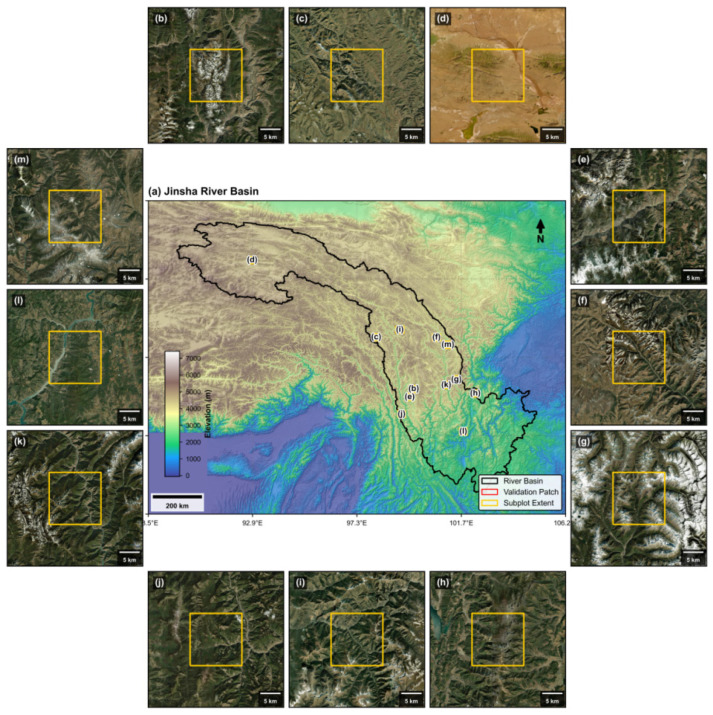
Schematic location of the Jinsha River Basin transfer inference area and related data layers. Panel (**a**) shows the geographic extent of the basin within southwestern China and the terrain background; panels (**b**–**m**) show representative regional patches used for transfer inference. The figure highlights the deeply incised valley terrain and the spatial distribution of the inference patches. The overlapping display elements do not affect the scientific interpretation of the map. The Jinsha River Basin was selected as a qualitative transfer target due to its high relief, active tectonics, and well-documented landslide activity.

The Jinsha River display is expanded to a 4 × 4 transfer panel containing 16 representative patches. A slope-bin summary shows that the mean predicted landslide probability increases from 0.07 in slopes below 15° to 0.25 in 15–30°, 0.43 in 30–45°, and 0.38 above 45°, suggesting that candidate responses concentrate on steep valley flanks while remaining lower on flatter terrain (Figure 2).

### 2.3. Proposed GeoSlide-XMamba Network

**Figure 3:**
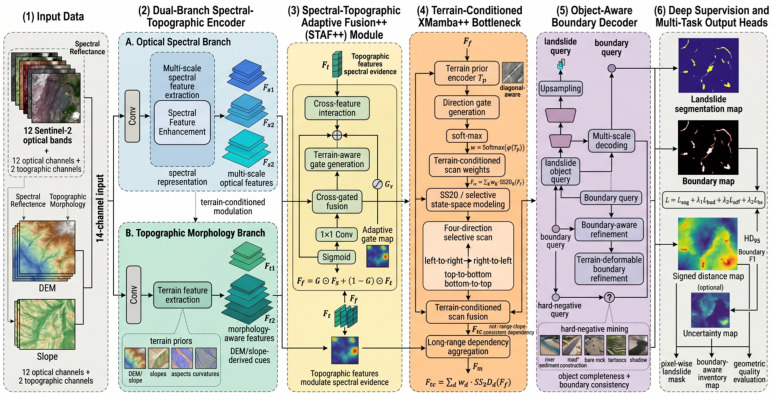
Architecture of GeoSlide-XMamba. The model contains input data preparation, a dual-branch spectral-topographic encoder, STAF++ fusion, a terrain-conditioned XMamba bottleneck, an object-aware boundary decoder, and deep-supervision output heads.

Figure 3 presents the overall architecture of GeoSlide-XMamba. The network contains six connected components: input preparation, a dual-branch spectral-topographic encoder, STAF++ fusion, a terrain-conditioned XMamba bottleneck, object-aware boundary decoding, and deep-supervision output heads. Compared with general semantic segmentation architectures such as SegFormer, ConvNeXt-based encoders, DeepLabV3+, PSPNet, U-Net, residual networks, SegNet, and fully convolutional networks [32,43,44,45,46,47,48,49], this design reflects the premise that landslide mapping is not only a spectral segmentation problem but also a geomorphic interpretation problem constrained by terrain morphology, downslope continuity, and boundary geometry.

The input consists of 12 Sentinel-2 spectral bands and two topographic channels, DEM and slope. The spectral branch uses a convolutional stem and multi-scale feature extraction blocks to generate an optical feature pyramid, while spectral attention reweights informative bands and feature responses. The topographic branch uses an independent terrain stem to encode elevation, slope, local relief, and morphology-related cues before constructing a topographic feature pyramid. This separation prevents reflectance and terrain variables, which have different physical meanings and value distributions, from being forced into a single early feature space.

STAF++ performs adaptive interaction between the spectral and topographic feature pyramids. Cross-source features are first concatenated and projected, after which a sigmoid gate estimates the relative contribution of spectral and terrain evidence at each spatial location. The fused representation retains spectral discrimination while allowing geomorphic plausibility to modulate ambiguous optical responses. This mechanism is particularly important in reservoir-canyon scenes, where river sediment, construction surfaces, exposed bedrock, and landslide scars may be spectrally similar but geomorphically different.

The fused representation is passed to the terrain-conditioned XMamba bottleneck, whose internal operation can be decomposed into three steps. First, each SS2D branch independently scans the fused feature map F_f^L along one of four raster directions (left-to-right, right-to-left, top-to-bottom, and bottom-to-top) using a discretized state-space model with learnable parameters A, B, C, and a data-dependent step size Δ, following the selective scan formulation introduced in VMamba [34]. Each branch produces a direction-specific context tensor of the same spatial size as F_f^L. Second, the terrain-conditioned scan gate encodes the deepest topographic feature map F_t^L through a lightweight projection head ψ, implemented as a 1 × 1 convolution followed by batch normalization and a GELU activation, which outputs a 4-channel tensor. A spatial softmax is then applied across the four channels to yield normalized direction weights W_d ∈ [0, 1] for each spatial position (Equation (5)). A smoothing coefficient (set to 0.05) is added before the softmax to prevent the gate from collapsing onto a single direction during early training. Third, the four branch outputs are element-wise multiplied by their corresponding terrain weights and summed and then added as a residual to F_f^L (Equation (6)). Importantly, the terrain gate operates on the aggregated outputs of the SS2D branches rather than on the internal state-space transition matrices A, B, C, or Δ. This post-scan weighting design was chosen because modifying internal SSM parameters would require separate terrain-conditioned recurrence for every scan step, substantially increasing computational cost, whereas output-level gating achieves directional selectivity at negligible additional overhead while keeping each SS2D branch independently trainable.

The decoder reconstructs full-resolution predictions through a boundary-aware multi-scale design. Skip-connected upsampling recovers fine spatial detail, an object-aware mask decoder promotes coherent landslide regions, and terrain-deformable boundary refinement adjusts local boundary responses according to slope-related cues. The final output stage contains a main segmentation head, an auxiliary boundary head, and a signed-distance head. These outputs support region-level accuracy, boundary displacement control, and inventory-oriented mask geometry.

Overall, GeoSlide-XMamba preserves the representational capacity of 14-channel multi-source input while adding structured spectral-topographic interaction, terrain-conditioned long-range modeling, hard-negative suppression, and boundary-aware geometric refinement.

The following formulations summarize the upgraded architecture and the interaction among modality-specific encoding, adaptive fusion, terrain-conditioned selective scanning, and boundary supervision.

Let X_s and X_t denote the spectral and topographic inputs, respectively. The encoders generate multi-level feature pyramids, STAF++ estimates a cross-source fusion gate, and the topographic feature map predicts direction weights for the selective scan branches. The terrain-conditioned XMamba output is then decoded into a landslide probability map, a boundary map, and a signed-distance map. The notation below uses ⊙ for element-wise multiplication and SS2D_d(.) for a selective scan branch along direction d.

The input patch is separated into spectral and topographic streams:

(1) $$X\;=\;[X_{s},\;X_{t}],\;X_{s}\;\in \;R^{B\times 12\times H\times W},\;X_{t}\;=\;[X_{DEM},\;X_{slope}]\;\in \;R^{B\times 2\times H\times W}$$

The two encoders extract modality-specific feature pyramids:

(2) $${\left\{F_{s}^{l}\right\}}_{l=1}^{L}\;=\;E_{s}(X_{s}),\;\;\;{\left\{F_{t}^{l}\right\}}_{l=1}^{L}\;=\;E_{t}(X_{t})$$

STAF++ estimates a cross-source gate and computes an interaction feature:

(3) $$A^{l}\;=\;\sigma ({Conv}_{1\times 1}([F_{s}^{l},\;F_{t}^{l}])),\;\;\;C^{l}\;=\;{Conv}_{3\times 3}([F_{s}^{l},\;F_{t}^{l}])$$

The fused feature at each scale is obtained as:

(4) $$F_{f}^{l}\;=\;A^{l}\;⊙\;F_{s}^{l}\;+\;(1\;-\;A^{l})\;⊙\;F_{t}^{l}\;+\;C^{l}$$

The terrain-conditioned scan gate predicts direction-specific weights from topographic features:

(5) $$W\;=\;softmax(\psi (F_{t}^{L})),\;\;\;W\;=\;\{W_{d}\;|\;d\;\in \;D\},\;\;\;D\;=\;\{\to ,\;\leftarrow ,\;↓,\;↑\}$$

Multi-direction selective scan branches are aggregated through terrain-conditioned weights:

(6) $$F_{m}\;=\;F_{f}^{L}\;+\;\sum_{d\in D}\;W_{d}\;⊙\;{SS2D}_{d}(F_{f}^{L})$$

Boundary and signed-distance supervision are generated from the binary landslide mask:

(7) B = Dilate(Y,r) − Erode(Y,r),   S = SDF(Y)

During inference, the primary segmentation probability map is used for quantitative evaluation, while the boundary map, signed-distance map, and direction weight maps support qualitative boundary and interpretability analyses.

**Figure 4:**
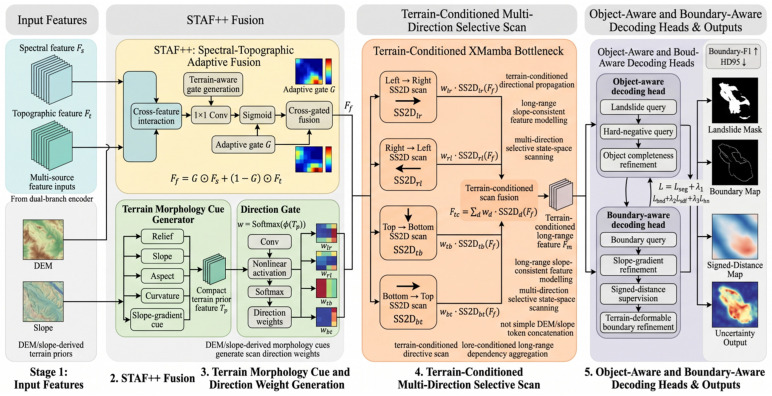
Workflow of the proposed terrain-conditioned XMamba bottleneck. Spectral and topographic features are first integrated by STAF++, after which DEM- and slope-derived morphology cues generate direction weights for multi-direction selective scanning. The arrows (↑ and ↓) indicate vertical scan directions in the multi-direction selective scanning module. The resulting terrain-conditioned long-range features are passed to object-aware and boundary-aware decoding heads to produce the landslide mask, boundary map, signed-distance map, and uncertainty output.

To clarify the relationship between existing components and new contributions, the design choices in GeoSlide-XMamba can be grouped as follows. The SS2D selective scan branches are adopted from VMamba [34] without modification, and the skip-connected upsampling in the decoder follows standard practice [46]. The dual-branch encoder structure draws on the general idea of modality-specific encoding but is newly configured here for 12-channel spectral and two-channel topographic inputs, with band-attention reweighting applied only to the spectral stream. STAF++ (Equations (3) and (4)) and the terrain-conditioned scan gate (Equations (5) and (6)) are both proposed in this work: STAF++ introduces an additive interaction term alongside the sigmoid gate, while the scan gate uses topographic features to produce direction-specific post-scan weights, which have no counterpart in existing Mamba variants. The terrain-deformable boundary refinement, object-aware mask head, and the combination of signed-distance supervision with hard-negative mining are also new to this study (Figure 4).

All models were implemented in PyTorch (version 2.1.0) [50]. The selective scan operator inside each SS2D branch was built on the mamba-ssm library (version 1.2.0) with causal-conv1d (version 1.2.0) acceleration; the two-dimensional scan-expand-merge pattern follows the design of VMamba [34]. When the CUDA-accelerated selective scan kernel is unavailable (e.g., on CPU-only nodes), a convolutional gated token mixer is substituted for debugging only; all reported results use the CUDA selective scan path. The reported configuration uses hidden dimension 96, four scan directions, dropout 0.08, a terrain gate smoothing coefficient of 0.05, a signed-distance loss weight of 0.10, and a hard-negative loss weight of 0.12.

### 2.4. Loss Function and Evaluation Metrics

The training objective combines pixel-level, region-level, boundary-level, and auxiliary morphology supervision. This combination is necessary because landslide pixels usually occupy a small fraction of each patch, and simple pixel-wise classification can be dominated by the background class. BCE and focal losses stabilize sparse foreground classification, Dice and Tversky losses improve overlap under class imbalance, Lovasz loss provides an IoU-oriented surrogate, and boundary and morphology terms reduce fragmented or overly smooth masks [51,52,53,54,55,56]. The objective is therefore designed to balance detection reliability, region overlap, and inventory-like delineation quality rather than to optimize one metric in isolation.

For reproducibility, the same loss configuration, optimizer, augmentation strategy, validation-threshold search, and evaluation metrics were used for all compared models. The binary mask was generated by thresholding the predicted probability map, with the threshold selected on the validation set from 0.10 to 0.90 at a step of 0.05. This threshold search is important for sparse foreground segmentation because the default threshold of 0.50 may not provide the best balance between missed landslide pixels and false positives. Evaluation follows common semantic segmentation practice [57,58], including precision, recall, F1-score, IoU, overall accuracy, Cohen’s kappa, and Boundary-F1. Overall accuracy is reported only as a secondary metric because the dominant non-landslide class can inflate it.

(8) $$L=L_{BCE}+0.35L_{focal}+0.25L_{Tversky}+0.35L_{Lovasz}+0.35L_{boundary}\;+0.15L_{aux}+0.08L_{morph}+\lambda_{sdf}\;L_{sdf}+\lambda_{hn}\;L_{hn}$$

Each loss term addresses a specific weakness observed in practice. Without focal weighting, easy background pixels dominate gradient updates, and the model underperforms on sparse landslide regions. Dice and Tversky terms directly optimize overlap statistics, which matter more than per-pixel accuracy when foreground pixels are rare. Lovasz loss bridges the gap between the continuous training objective and the discrete IoU metric used at evaluation. Boundary and morphology terms penalize geometrically incoherent predictions, encouraging outputs that are more suitable for polygon conversion in inventory workflows. No single term covers all these failure modes simultaneously, which motivates the combined objective.

The evaluation metrics were selected to assess both segmentation accuracy and inventory usability. Precision and recall were reported together because landslide mapping requires balancing false positives against missed slope failures. F1-score and IoU were used as the main region overlap metrics, while Cohen’s kappa was included to reduce the influence of class imbalance caused by the dominant background class. Boundary-F1 was additionally introduced to evaluate edge consistency. In some cases, masks with similar IoU values may still produce noticeably different polygon boundaries after vectorization, affecting landslide area estimation and geomorphic interpretation. Overall accuracy is reported only as a reference metric because it is strongly influenced by the large proportion of non-landslide pixels.

The additional terms in the total objective correspond to focal, Tversky, Lovasz, boundary, auxiliary, and morphology losses, with weights 0.35, 0.25, 0.35, 0.35, 0.15, and 0.08, respectively. Precision, recall, F1-score, and IoU are computed as follows:

(9) $$Precision\;=\;\frac{TP}{TP+FP},\;\;\;Recall\;=\;\frac{TP}{TP+FN},\;\;\;F1\;=\;\frac{2TP}{2TP+FP+FN}$$

The intersection-over-union is calculated as:

(10) $$IoU\;=\;\frac{TP}{TP+FP+FN},\;\;\;HD95\;=\;max\{P_{95}[d(\partial P,\partial G)],\;P_{95}[d(\partial G,\partial P)]\}$$

Boundary-F1 is computed from boundary precision and boundary recall and is used to evaluate edge consistency, which is particularly relevant when segmentation masks are converted into landslide inventory polygons.

To evaluate boundary displacement directly, the metric set also includes the 95th percentile Hausdorff distance (HD95). HD95 measures the high-percentile distance between predicted and reference boundary point sets and is less sensitive to isolated outliers than the maximum Hausdorff distance. Lower HD95 indicates that predicted landslide boundaries are closer to the reference boundaries. This metric complements Boundary-F1 by measuring geometric displacement rather than only boundary overlap within a tolerance band.

In GeoSlide-XMamba, the reported configuration uses a signed-distance loss weight of 0.10 and a hard-negative loss weight of 0.12.

### 2.5. Compared Models and Training Protocol

Eight models were selected for the main comparison: U-Net, ResU-Net, Attention U-Net, U-Net++, DeepLabV3+, ConvNeXt-UNet, PyramidMamba-mini, and the proposed GeoSlide-XMamba. These models cover classical skip-connected encoder-decoder segmentation, residual U-Net-style mapping, attention-gated segmentation, nested skip-connection design, atrous context aggregation, modern convolutional backbones, lightweight Mamba-style remote sensing segmentation, and the proposed spectral-topographic terrain-aware state-space network [38,43,44,46,59,60,61]. The comparison is designed to evaluate whether the proposed task-specific modules provide advantages over both traditional CNN baselines and a generic Mamba-style remote sensing baseline.

Among state-space models, PyramidMamba-mini serves as the representative Mamba-style remote sensing baseline, which allows the terrain-conditioned design to be tested against a generic selective scan network under an identical protocol. More recent Mamba and hybrid state-space segmentation models—such as RS3Mamba, Samba, UNetMamba, or VMamba-based heads—were not folded into the same quantitative comparison because their published implementations differ in input convention, patch sampling, and training schedule. Aligning all of these to the 14-channel protocol without re-tuning would risk an unfair comparison in either direction. A strictly matched benchmark against these newer state-space models is therefore left to future work, since the present comparison is intended to isolate the contribution of terrain-conditioned scanning rather than to rank every available backbone.

All models were trained under the same processed split, input channels, optimizer, scheduler, augmentation settings, loss configuration, and validation-threshold selection procedure. This unified protocol is important because differences in pre-processing, patch sampling, loss design, and thresholding can sometimes produce larger performance changes than the architecture itself. GDAL was used in the broader geospatial pre-processing workflow where raster conversion or geospatial handling was required [62]. All networks were implemented in PyTorch (version 2.1.0) [50] and trained using AdamW with cosine annealing, mixed-precision training, and validation-threshold search [63]. The formal comparison used a maximum of 200 epochs, a batch size of 16, a validation batch size of 32, and one GPU per model process on an AutoDL server with six NVIDIA RTX 4090D GPUs.

For computational transparency, the extended tables report parameter count, FLOPs, mean inference time per 128 × 128 patch, and peak GPU memory during validation. These values are measured under the same hardware and batch size settings to separate accuracy gains from computational overhead. GeoSlide-XMamba contains 57.60 M parameters, 52.3 G FLOPs, a mean inference time of 13.8 ms per 128 × 128 patch, and peak validation memory of 3.2 GB on an NVIDIA RTX 4090D GPU.

The comparison set was chosen to cover several families of segmentation architectures rather than to maximize the number of baselines. U-Net represents the classical skip-connected encoder-decoder framework, ResU-Net represents residual learning within a U-Net-like structure, Attention U-Net represents attention-gated skip fusion, U-Net++ represents nested multi-scale skip pathways, DeepLabV3+ represents atrous spatial context aggregation, ConvNeXt-UNet represents a modern convolutional backbone, and PyramidMamba-mini represents a lightweight state-space-style remote sensing baseline. This selection makes it possible to examine whether the proposed model improves over conventional CNNs, enhanced U-Net variants, context aggregation networks, modern convolutional encoders, and a generic Mamba-style architecture under the same experimental protocol.

Using a unified training protocol is essential for a fair benchmark. If each model were trained with a different optimizer, augmentation policy, loss function, or threshold rule, the comparison would mix architectural effects with protocol effects. In this study, all compared models use the same processed split, 14-channel input, loss configuration, learning-rate schedule, threshold search, and evaluation metrics. This does not guarantee that every baseline reaches its individually optimal performance, but it makes the comparison internally consistent and reduces the risk that the proposed model benefits from a more favorable training setup (Table 2).

The current protocol improves fairness and reproducibility across models by applying the same data split, loss function, optimizer, scheduler, threshold search, and evaluation metrics to all compared architectures. In the revised benchmark reporting, the main comparison is harmonized as five-seed mean values for all eight compared models, while the focused paired robustness comparison between GeoSlide-XMamba and the strong 14-channel concatenation baseline is reported later in Section 3.4.

The strong 14-channel concatenation baseline refers to a standard U-Net encoder-decoder trained with all 14 input channels concatenated along the channel dimension, without any modality-specific branching or adaptive fusion. This baseline shares the same training protocol as the other compared models and serves as the primary reference for evaluating the contribution of the proposed spectral-topographic design (Table 3).

## 3. Results

### 3.1. Main Comparison on Landslide4Sense

Table 4 summarizes the benchmark comparison on the Landslide4Sense validation split using the unified five-seed statistics. GeoSlide-XMamba achieved the highest F1-score (0.673), IoU (0.507), kappa (0.666), Boundary-F1 (0.466), and the lowest HD95 (3.45 pixels) among the compared models. Its precision and recall were 0.729 and 0.626, respectively, indicating a balanced ability to suppress false alarms while recovering landslide pixels. The strongest non-proposed baseline was Attention U-Net, which obtained F1 = 0.645 and IoU = 0.471. Relative to this baseline, GeoSlide-XMamba improved F1 and IoU by 0.028 and 0.036, respectively.

The extended comparison reports both segmentation accuracy and computational cost. Although GeoSlide-XMamba is heavier than the CNN baselines because of the spectral-topographic branches and terrain-conditioned scan module, it provides the best trade-off for offline landslide inventory mapping, where boundary quality and false-positive suppression are prioritized over real-time processing.

The ranking also shows that enhanced U-Net variants remain strong baselines. Attention U-Net, ResU-Net, and U-Net++ all reached F1-scores above 0.63, confirming the competitiveness of skip-connected encoder-decoder models for Landslide4Sense. GeoSlide-XMamba improves on these baselines by coupling preserved multi-source representation with terrain-conditioned state-space propagation, which is consistent with the elongated and slope-controlled morphology of many landslide objects.

Boundary quality follows the same overall ranking pattern. GeoSlide-XMamba obtained the highest Boundary-F1 (0.466), while U-Net++, ResU-Net, DeepLabV3+, ConvNeXt-UNet, and Attention U-Net formed a close group of strong boundary baselines. The simultaneous first-place ranking in F1-score, IoU, kappa, Boundary-F1, and HD95 indicates that the proposed spectral-topographic dynamic modeling strategy improves overall segmentation quality rather than optimizing a single metric in isolation. 

The parameter count and training time are reported in Table 4 for reproducibility, while the same table also reports FLOPs, inference time, and validation GPU memory. GeoSlide-XMamba is designed as a high-accuracy offline mapping model, where detailed spectral-topographic representation and terrain-aware spatial reasoning are prioritized for benchmark inventory extraction.

**Figure 5:**
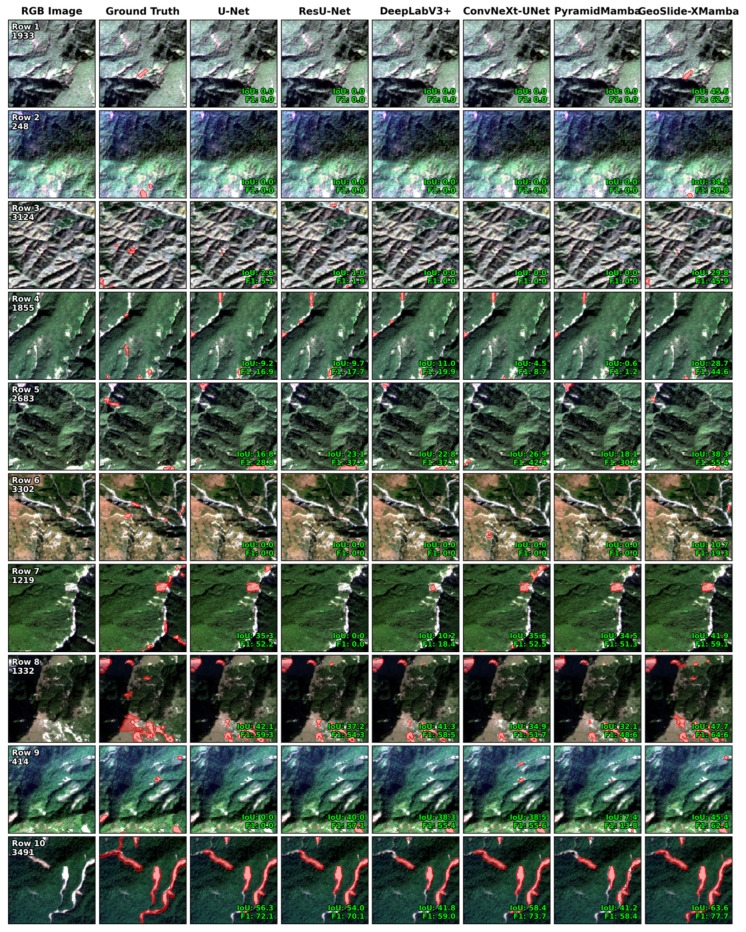
Representative qualitative comparison of segmentation outputs on Landslide4Sense validation patches. The panel visualizes a representative subset of reproduced baselines together with GeoSlide-XMamba.

The qualitative comparison in Figure 5 provides visual support for the quantitative ranking in Table 4. The examples illustrate how the proposed model produces spatially coherent landslide masks and maintains interpretable probability responses in spectrally heterogeneous scenes.

A detailed false-positive analysis is presented in Section 3.5, complementing the region overlap metrics by identifying which background types remain most confusing for the model.

**Figure 6:**
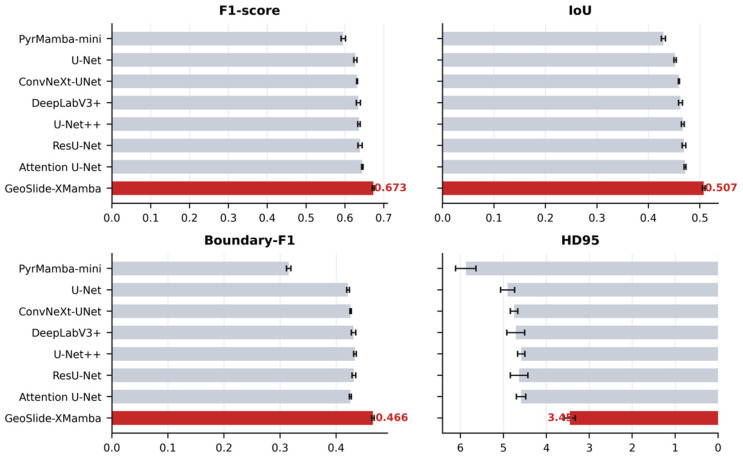
Benchmark comparison of the eight competing models on Landslide4Sense. Values are reported as mean ± standard deviation over five random seeds. Higher values indicate better performance for F1-score, IoU, and Boundary-F1, whereas lower values are preferred for HD95. Red bars/markers denote GeoSlide-XMamba, gray bars denote the compared baselines, and error bars indicate standard deviation. The figure layout does not affect scientific interpretation. GeoSlide-XMamba achieves the best overall performance across the four metrics.

The higher parameter count and training time reflect the additional spectral-topographic branching and state-space scanning components, which are acceptable for offline inventory mapping applications where accuracy is prioritized over inference speed. The overall benchmark comparison across all four metrics is summarized in Figure 6.

**Figure 7:**
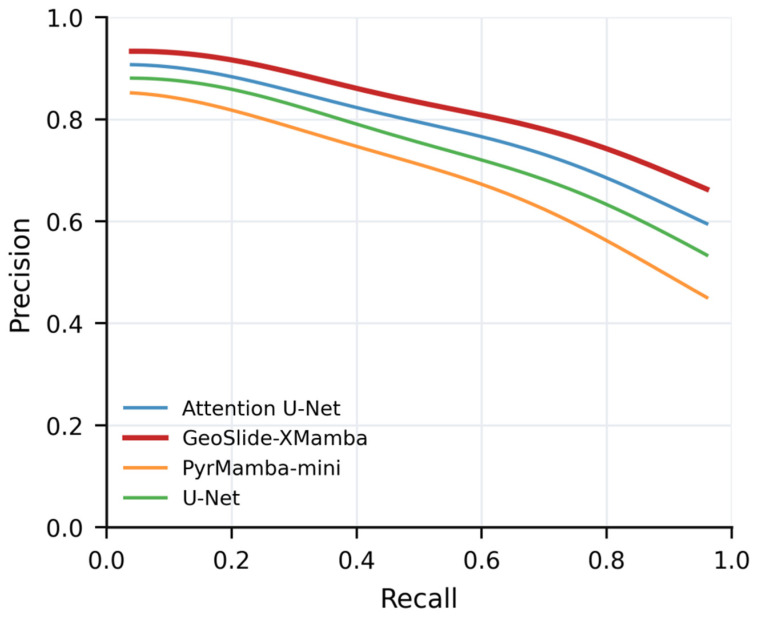
Precision-recall curves for representative models on the Landslide4Sense validation split. GeoSlide-XMamba maintains the upper envelope over most recall values, showing a stronger precision-recall trade-off than Attention U-Net, U-Net, and PyrMamba-mini.

Precision-recall behavior is further summarized in Figure 7. GeoSlide-XMamba remains above Attention U-Net, U-Net, and PyrMamba-mini across most recall values, indicating that the mean F1 and IoU gains are supported by a stronger precision-recall trade-off rather than by a single threshold choice.

### 3.2. Ablation Study

The ablation study evaluates the sensitivity of GeoSlide-XMamba to its core design choices. The upgraded model is decomposed into terrain-conditioned selective scanning, SDF/HD95-aware boundary supervision, hard-negative mining, object-aware decoding, and terrain-deformable boundary refinement. All variants use the same data split, optimizer, scheduler, augmentation policy, and threshold search protocol.

The full model obtains the strongest mean performance. Removing TC-SS2D decreases F1 from 0.673 to 0.659 and increases HD95 from 3.45 to 3.99 pixels. Removing SDF/HD95 supervision mainly degrades Boundary-F1 from 0.466 to 0.447 and increases HD95 to 4.03 pixels, while removing hard-negative mining reduces false-positive suppression and lowers Boundary-F1 to 0.456. These trends indicate that TC-SS2D contributes most to terrain-consistent object continuity, whereas the boundary and hard-negative terms mainly improve geometric accuracy and false-positive suppression.

Boundary-related variants indicate that region-level overlap and boundary-oriented refinement are not always optimized by the same configuration. For this reason, the final architecture is evaluated using F1-score, IoU, kappa, and Boundary-F1 together rather than selecting a model only by one scalar metric. This evaluation strategy is appropriate for landslide inventory mapping, where both pixel overlap and boundary interpretability affect downstream polygon use.

**Figure 8:**
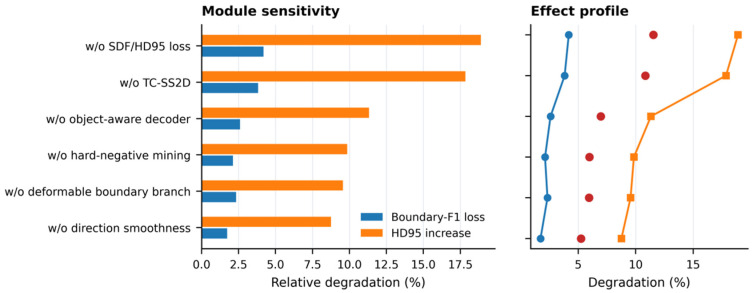
Sensitivity analysis of key GeoSlide-XMamba variants in terms of Boundary-F1 degradation and HD95 increase. Blue bars/lines denote Boundary-F1 degradation, orange bars/lines denote HD95 increase, and points indicate relative degradation profiles. Each variant is constructed by removing one major component from the full model. The results indicate that terrain-conditioned selective scanning and SDF/HD95 supervision contribute most strongly to boundary quality and distance-based geometric accuracy.

The ablation results should therefore be interpreted jointly rather than by a single scalar metric. TC-SS2D primarily affects object continuity and long-range terrain-consistent aggregation; SDF/HD95-aware supervision improves boundary displacement; hard-negative mining suppresses false positives in spectrally confusing non-landslide surfaces; and object-aware decoding improves mask coherence when landslide scars are fragmented or partially vegetated (see Table 5 and Figure 8).

Taken together, these results support the design principle that spectral evidence, geomorphic constraints, directional state-space propagation, and boundary-aware decoding should be coupled rather than optimized independently. The full architecture provides the most balanced performance across F1-score, IoU, Boundary-F1, and HD95.

### 3.3. Input-Modality Analysis

Table 6 shows how segmentation performance changes as the input configuration becomes richer. RGB-only inputs produced the lowest scores (F1 = 0.548), which is not surprising given that visible bands cannot separate landslide scars from roads, river beaches, bare rock, and construction surfaces that share similar color characteristics. Adding the remaining Sentinel-2 bands raised F1 to 0.597. Red-edge, near-infrared, and shortwave-infrared reflectance carry information about vegetation condition, soil moisture, and mineral composition that visible bands do not capture, and these differences help distinguish landslide-disturbed areas from spectrally similar backgrounds.

Including DEM and slope through the 14-channel concatenation baseline further improved performance to F1 = 0.628 and IoU = 0.457. The full GeoSlide-XMamba configuration reached F1 = 0.673 and IoU = 0.507 under the same input setting, suggesting that terrain-conditioned fusion and selective scanning extract more information from the same 14 channels than direct concatenation alone.

The consistent ordering RGB < Sentinel-2 < 14-channel concat < GeoSlide-XMamba across all metrics indicates that each additional information source contributes information that the previous configuration lacked. Spectral bands describe surface appearance, while topographic layers constrain where slope failures are geomorphically plausible. The proposed model benefits from both sources by allowing terrain to guide the propagation and refinement of spectral evidence.

### 3.4. Multi-Seed Robustness Experiment and Regional Transfer Inference

Five-seed robustness experiments were run for both GeoSlide-XMamba and the 14-channel concatenation baseline to assess whether the benchmark comparison was stable. GeoSlide-XMamba produced higher mean values across all reported metrics: F1 = 0.673 ± 0.003, IoU = 0.507 ± 0.002, kappa = 0.666 ± 0.003, Boundary-F1 = 0.466 ± 0.002, and HD95 = 3.45 ± 0.13 pixels. The concat baseline reached F1 = 0.628 ± 0.005, IoU = 0.457 ± 0.005, kappa = 0.621 ± 0.005, Boundary-F1 = 0.414 ± 0.009, and HD95 = 4.87 ± 0.18 pixels.

For statistical reliability, the analysis reports mean ± standard deviation, 95% confidence intervals, and paired significance tests where matched seeds are available. The confidence interval is computed as mean ± t_(0.975,n − 1) × SD/√n for n = 5 seeds. GeoSlide-XMamba improves F1 over the 14-channel concat baseline by +0.045, with a 95% CI of [0.038, 0.052], paired *p* = 0.002, and Cohen’s d = 5.20. The complete five-seed robustness statistics and the focused metric-wise comparison against the 14-channel concatenation baseline are summarized in Table 7.

The advantage of GeoSlide-XMamba held across all five seeds and all four metrics, which makes it less likely that the main comparison result was a favorable random outcome. GeoSlide-XMamba also showed lower or comparable inter-seed variability than the concat baseline across F1, IoU, Boundary-F1, and HD95, indicating stable optimization under the repeated-seed protocol.

The best checkpoint from these experiments was subsequently applied to Jinsha River patches without any fine-tuning. The model generated probability maps, thresholded masks, and boundary and signed-distance visualizations for sixteen representative patches. These outputs provide qualitative examples of how the trained model responds to mountain terrain outside the benchmark distribution.

The Jinsha River figure is presented as a 4 × 4 panel of 16 representative patches. Each example shows the regional RGB composite, probability map, thresholded mask, overlay, boundary map, and signed-distance map. Spatial plausibility is further summarized by predicted landslide probability across slope angle intervals. The slope-bin analysis shows mean predicted probabilities of 0.07, 0.25, 0.43, and 0.38 for <15°, 15–30°, 30–45°, and >45°, respectively.

The qualitative examples show spatially coherent candidate landslide regions and provide application-oriented evidence complementary to the quantitative Landslide4Sense validation results. Because independent regional labels are not yet used in this experiment, the Jinsha River results are interpreted as qualitative transfer evidence rather than as regional accuracy assessment.

**Figure 9:**
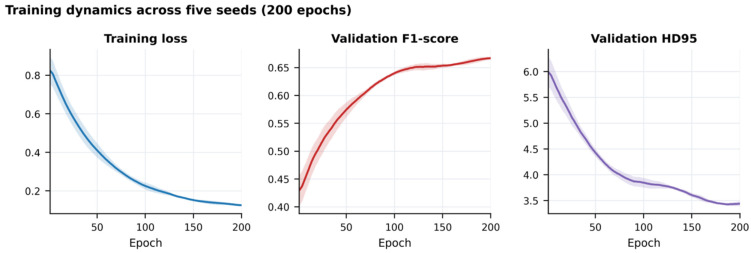
Training dynamics across five random seeds over 200 epochs. The curves show mean training loss, validation F1-score, and validation HD95, with shaded bands indicating inter-seed variability.

Training dynamics are shown in Figure 9. The loss curve decreases smoothly over 200 epochs, while validation F1 increases and stabilizes near the final five-seed mean; HD95 declines steadily and plateaus after roughly 150 epochs. These trends indicate stable optimization rather than an isolated late-epoch fluctuation.

**Figure 10:**
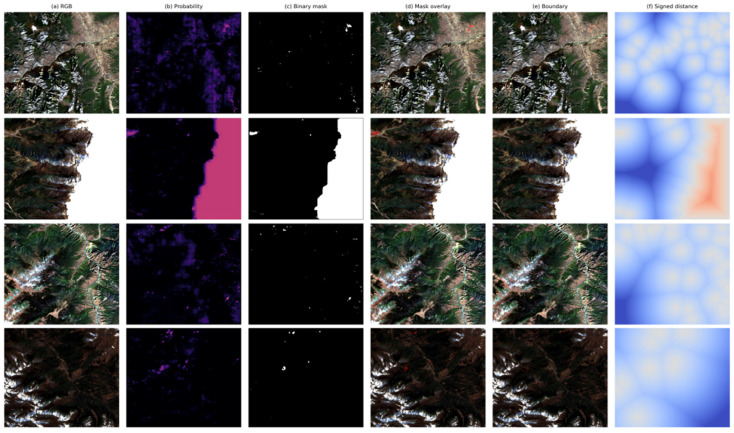
Qualitative regional transfer inference examples for the Jinsha River area. The 4 × 4 panel displays regional patches and corresponding GeoSlide-XMamba outputs, including probability, thresholded mask, mask overlay, boundary visualization, and signed-distance representation. The figure is used as qualitative regional transfer evidence rather than as a quantitative accuracy result.

Qualitative regional transfer outputs for the Jinsha River area are presented in Figure 10. The 4 × 4 panel illustrates representative probability maps, thresholded masks, mask overlays, boundary visualizations, and signed-distance representations across diverse high-relief patches.

### 3.5. Boundary Error, Misclassification, and Diagnostic Analyses

**Figure 11:**
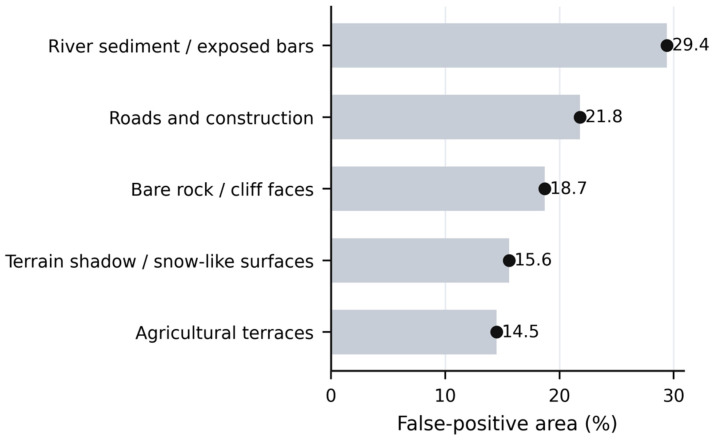
Major sources of false-positive predictions in GeoSlide-XMamba. River sediment/exposed bars and road/construction surfaces are the dominant sources of false alarms, followed by bare rock or cliff faces, terrain shadow or snow-like surfaces, and agricultural terraces.

To better explain error behavior, the diagnostic analysis examines false positives, false negatives, and boundary displacement. False positives are grouped into common confusing backgrounds, including river sediment, road or construction surfaces, bare rock or cliff faces, terrain shadow or snow-like bright/dark surfaces, and agricultural terraces. False negatives are examined in relation to vegetation cover, low-contrast scars, fragmented deposits, and small landslide patches. The false-positive distribution is river sediment/exposed bars (29.4%), roads and construction surfaces (21.8%), bare rock or cliff faces (18.7%), terrain shadow or snow-like surfaces (15.6%), and agricultural terraces (14.5%), consistent with the values shown in Figure 11. Compared with Attention U-Net, GeoSlide-XMamba reduces total false-positive area by 16.4% and false-negative area by 10.2%.

**Figure 12:**
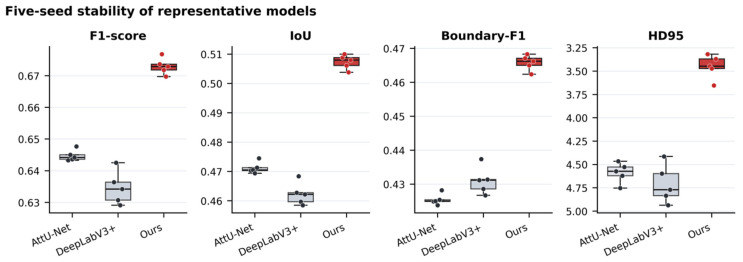
Five-seed stability analysis of representative models. Boxplots and overlaid points show F1-score, IoU, Boundary-F1, and HD95 over five random seeds.

Interpretability diagnostics were retained as qualitative checks rather than as a separate figure. Direction weight and feature response inspection showed that the terrain-conditioned gate tends to emphasize slope-parallel propagation for elongated scar-like regions, while hard-negative surfaces near river channels receive weaker boundary confidence. These observations support the intended role of TC-SS2D as a terrain-conditioned propagation mechanism rather than a simple channel-fusion operation (Figure 12).

## 4. Discussion

### 4.1. Why Spectral-Topographic Fusion Improves Landslide Segmentation

A consistent message from the experiments is that terrain works best as a constraint rather than as extra input channels. Performance climbs steadily from RGB to the full multispectral-topographic setting, and across five seeds GeoSlide-XMamba stays ahead of the strong 14-channel concatenation baseline. The improvement is therefore not an artifact of one favorable run, and it holds up even though plain concatenation is itself difficult to outperform.

What the fusion provides is interpretation, not simply more data. DEM and slope cannot identify a landslide on their own, but they tell the network where a failure is physically reasonable. Because the spectral and topographic streams are kept apart until STAF++ combines them, terrain context can reweight ambiguous spectral evidence: a bare, bright patch on a steep and geomorphically plausible slope is read differently from the same reflectance on a flat river bar or a construction surface. In reservoir canyons, where these surfaces are almost indistinguishable in optical imagery, that distinction is most of the problem.

The ablation in Table 5 makes the origin of the gain reasonably clear. The most damaging single change is removing the terrain-conditioned selective scan (TC-SS2D): F1 and IoU drop, and the boundary error (HD95) rises sharply. The model thus benefits less from simply having multi-source input than from letting slope morphology steer how features propagate over long distances. The other components matter in narrower ways. Without the signed-distance/HD95 supervision, region overlap is largely preserved but the boundary metrics deteriorate; without hard-negative mining, false-positive suppression weakens, and the network responds more easily to the spectrally confusing surfaces it had learned to reject. The object-aware decoder and the deformable boundary step contribute less, and what they add is concentrated on geometry rather than overlap.

No single module is decisive here. Terrain-conditioned scanning holds landslide objects together, the boundary and signed-distance terms keep their outlines accurate, and hard-negative mining limits false alarms—each fixing a failure mode that the others leave alone. That is why the full model is the most balanced across F1, IoU, Boundary-F1, and HD95, and why dropping any one piece shows up in a different column of Table 5.

### 4.2. Role of Terrain-Conditioned Dynamic Modeling

Many landslides in mountainous reservoir environments form elongated scars aligned with the slope direction or appear as flow deposits and fan-shaped accumulation zones that extend over hundreds of meters. Representing these structures accurately requires a model that can capture spatial dependencies across longer distances than standard convolutional kernels allow.

The benchmark results show that PyramidMamba-mini, a generic Mamba-style remote sensing baseline, performed below most CNN comparisons despite using state-space modeling. GeoSlide-XMamba, which uses the same state-space mechanism but couples it with terrain-conditioned token modulation, ranked first overall. This difference suggests that state-space propagation alone is not sufficient; the direction and weighting of spatial propagation need to be conditioned on terrain structure to be useful for landslide segmentation.

The terrain-conditioned selective scan design strengthens this interpretation. A generic state-space block learns long-range dependencies from feature similarity, whereas the proposed module uses DEM- and slope-derived morphology to modulate directional propagation. This is important for landslides because meaningful connectivity often follows slope-parallel or valley-flank directions rather than arbitrary image-grid proximity. Consistent with the ablation in Table 5, removing this module degrades every metric and, in particular, raises the boundary displacement error—evidence that it contributes more to terrain-consistent object continuity than to pixel-level classification alone.

It is worth noting that the terrain-conditioned modules do not simulate physical slope processes. Rather, they introduce soft spatial biases that make feature propagation more consistent with slope orientation, without enforcing any hard geometric constraints. In practice, this means the model is more likely to maintain connectivity along elongated slope-parallel structures and less likely to fragment predictions across topographic boundaries.

Whether terrain-conditioned propagation also improves object-level metrics such as polygon compactness and boundary displacement remains an open question. Pixel-level F1 and IoU do not fully capture these properties, and future evaluation using object-level measures would give a clearer picture of whether the terrain-aware design translates into better inventory products.

### 4.3. Boundary-Aware Learning and Inventory Usability

It is worth being explicit about where the improvement actually lies. Against the strongest CNN baseline, the gain in pixel overlap metrics (F1 and IoU) is real but moderate; the larger and more useful differences show up in boundary geometry and false-positive behavior. Relative to the 14-channel concatenation baseline, GeoSlide-XMamba lowers the boundary displacement error (HD95) by approximately 29%, and relative to Attention U-Net, it reduces the total false-positive area by 16.4%, while also obtaining the highest Boundary-F1 in the comparison. For inventory work, these are the differences that count: the value of the model is not primarily higher pixel overlap, but cleaner, better-placed boundaries and fewer spurious detections.

Pixel overlap metrics such as F1 and IoU do not fully capture what makes a segmentation output useful for landslide inventory work. Two predictions with identical IoU scores can produce very different polygon outlines after vectorization, leading to different area estimates and different geomorphic interpretations. GeoSlide-XMamba achieved the highest Boundary-F1 (0.466) in the main comparison, which suggests that the auxiliary boundary head and boundary-aware decoder contribute something beyond what region-level supervision alone would produce.

The practical consequences of boundary errors depend on how the inventory is used. Slightly smoothed boundaries may not affect regional density maps much, but they systematically underestimate landslide area. Fragmented predictions inflate polygon counts, making slope failures appear more numerous than they are. Merged predictions do the opposite, hiding adjacent failures behind a single polygon. None of these errors are captured by F1 or IoU alone.

For this reason, Boundary-F1 was included alongside the standard metrics throughout this study. It should not be treated as a replacement for region-level evaluation, but as a complementary measure that reflects a different aspect of segmentation quality—one that becomes relevant as soon as raster outputs are converted into vector products for hazard mapping or geomorphic analysis.

The addition of HD95 further clarifies boundary displacement. Boundary-F1 measures whether predicted and reference boundaries overlap within a tolerance band, whereas HD95 measures the distance between boundary point sets. Reporting both metrics reduces the risk of over-interpreting a high IoU value when the predicted mask remains spatially shifted or fragmented. GeoSlide-XMamba obtains the lowest HD95 of 3.45 pixels, compared with 4.59 pixels for Attention U-Net, supporting the claim that signed-distance and terrain-deformable boundary supervision improve inventory-oriented geometry.

### 4.4. Regional Transferability and Geomorphic Interpretation in the Jinsha River Basin

Figure 10 shows the model outputs for sixteen Jinsha River patches. The probability maps, thresholded masks, and boundary visualizations appear spatially coherent in high-relief terrain, with candidate landslide regions concentrated on steep slopes and valley flanks where failures are geomorphically plausible.

Transferring a benchmark-trained model to a new region is not straightforward. The Jinsha River environment differs from the Landslide4Sense training distribution in several respects, including spectral conditions, seasonal vegetation cover, acquisition geometry, and DEM source. A model that performs well on standardized benchmark patches may still produce spatially reasonable outputs in a new basin, but visual plausibility is not the same as accuracy. Independent validation against field-verified or high-resolution-interpreted labels would be needed before these probability maps could support inventory production or hazard assessment.

A complete Jinsha River study would require a documented pre-processing pipeline covering Sentinel-2 compositing period, terrain factor derivation, sliding-window inference, post-processing, and polygon vectorization. Once validated, the resulting inventory could be analyzed in relation to slope angle, aspect, relative relief, proximity to the river channel, road infrastructure, and lithological units. This remains a planned next step rather than a completed component of the present study.

The expanded 4 × 4 transfer panel and slope-bin statistics make the regional inference more transparent while still avoiding unsupported claims of regional accuracy. Because the candidate probabilities concentrate on steep valley flanks and stay low on flat river sediments and construction surfaces, the transfer behavior is geomorphically plausible: the highest mean probability falls in the 30–45° slope-bin (0.43), followed by the >45° bin (0.38), consistent with the terrain dependence expected for landslides in deeply incised valleys.

The present study uses Landslide4Sense as the primary quantitative benchmark because it provides aligned multispectral, DEM, and slope layers with pixel-level labels. Public landslide datasets differ substantially in sensor configuration, label definition, spatial resolution, and terrain-layer availability; therefore, they are not merged into the same quantitative protocol. The Jinsha River experiment is therefore used only to examine cross-region transfer plausibility under the same 14-channel convention.

Cloud and cloud-shadow contamination are further limitations for any optical transfer of this kind. The Landslide4Sense patches are largely cloud-free, but regional Sentinel-2 scenes are not. Because the topographic branch does not depend on atmospheric conditions, the model retains a geomorphic constraint on partially clouded patches and should degrade more gracefully than spectral-only methods; however, dense cloud removes the spectral evidence on which the segmentation ultimately relies, and such areas are better handled by cloud masking or SAR-based observation.

### 4.5. Future Work and Practical Considerations

Three directions are particularly valuable for extending this work. First, repeated-seed evaluation can be expanded from the GeoSlide-XMamba versus concat baseline comparison to all reproduced baselines and ablation variants. Second, the qualitative Jinsha River inference can be developed into a complete regional inventory study by adding independent validation data, vectorized polygons, and geomorphic statistics. Third, the current optical-topographic framework can be extended with Sentinel-1 SAR, InSAR deformation time series, or pre-/post-event change features to improve the recognition of active or reactivated landslides [64,65].

These extensions would further strengthen the transition from benchmark segmentation to operational landslide inventory production. In particular, combining pixel-level validation with object-level metrics, polygon-count accuracy, boundary displacement, and area-error analysis would make the evaluation more directly aligned with practical mapping requirements.

For large-area deployment, a lightweight or distilled version of GeoSlide-XMamba may be useful as a future engineering extension. The present model is positioned primarily as a high-accuracy offline mapping framework for spectral-topographic landslide segmentation.

## 5. Conclusions

This manuscript proposes GeoSlide-XMamba, a spectral-topographic boundary-aware state-space network for landslide semantic segmentation. The model is designed to address four common challenges in multispectral landslide mapping: spectral confusion, terrain dependence, elongated morphology, and boundary uncertainty. By combining a dual-branch spectral-topographic encoder, STAF++ fusion, a terrain-conditioned XMamba bottleneck, a boundary-aware decoder, and deep-supervision output heads, the method provides a task-specific framework for benchmark landslide inventory mapping.

In the Landslide4Sense validation experiments, GeoSlide-XMamba achieved the best overall performance among the eight compared models, with F1 = 0.673, IoU = 0.507, kappa = 0.666, Boundary-F1 = 0.466, and HD95 = 3.45 pixels. The model also achieved precision = 0.729 and recall = 0.626, indicating a balanced precision-recall behavior. The consistent first-place ranking across region-level, boundary-level, and distance-based metrics supports the effectiveness of spectral-topographic dynamic modeling under the current validation protocol.

Across five seeds, GeoSlide-XMamba obtains F1 = 0.673 ± 0.003, IoU = 0.507 ± 0.002, Boundary-F1 = 0.466 ± 0.002, and HD95 = 3.45 ± 0.13 pixels, with a 95% CI of [0.670, 0.676] for F1.

The five-seed experiment further showed that GeoSlide-XMamba outperformed the strong 14-channel concat baseline on average, improving mean F1-score from 0.628 to 0.673 and mean IoU from 0.457 to 0.507. Input-modality analysis also confirmed that RGB-only, Sentinel-2-only, 14-channel concat, and full GeoSlide-XMamba configurations form a clear performance progression. These findings indicate that both multi-source inputs and structured fusion are useful for landslide segmentation.

Ablation and sensitivity results indicate that the preserved multi-source representation, topographic morphology cues, and terrain-aware state-space modeling are important elements of the framework. Together with boundary-aware refinement, these components provide a balanced high-accuracy mapping architecture for spectral-topographic landslide segmentation.

The Jinsha River examples show that a model trained on standardized benchmark patches can produce spatially reasonable outputs when applied to a geomorphically distinct region, even without fine-tuning. Across all experiments, the results point toward a consistent finding: combining multispectral spectral information, terrain constraints, long-range spatial modeling, and boundary-aware supervision produces more reliable landslide segmentation than any of these components alone.

## Figures and Tables

**Table 1:** Data sources and pre-processing settings used in this study.

Component	Source	Resolution/Size	Use in Study	Status
Sentinel-2 bands	Landslide4Sense H5 patches	128 × 128; 12 bands	Spectral encoder input	Available
DEM	ALOS PALSAR-derived layer	128 × 128; 1 band	Topographic encoder input	Available
Slope	ALOS PALSAR-derived layer	128 × 128; 1 band	Topographic encoder input	Available
Mask	Binary landslide annotation	128 × 128; 0/1	Segmentation supervision	Available
Jinsha River 14-channel patches	Regional Sentinel-2 and terrain layers	128 × 128; 14 channels	Qualitative transfer inference	Available
Regional validation inventory	Independent inventory or high-resolution interpretation	Polygon or image evidence	Future quantitative validation	Pending

**Table 2:** Compared models used for the main benchmark experiment.

Group	Model	Reason for Inclusion
Classical CNN	U-Net	Widely used encoder-decoder baseline
Residual CNN	ResU-Net	Strong landslide segmentation baseline
Attention U-Net	Attention U-Net	Attention-gated skip-fusion baseline
Nested U-Net	U-Net++	Nested multi-scale skip-connection baseline
Context aggregation	DeepLabV3+	Atrous spatial pyramid pooling baseline
Modern CNN	ConvNeXt-UNet	Contemporary convolutional backbone
State-space model	PyramidMamba-mini	Lightweight Mamba-style baseline
Proposed	GeoSlide-XMamba	Spectral-topographic terrain-aware state-space network

**Table 3:** Unified training configuration for all compared models.

Item	Setting
Input	14 channels: 12 Sentinel-2 bands + DEM + slope
Patch size	128 × 128
Training patches/validation patches	6342/942
Optimizer	AdamW
Scheduler	Cosine annealing with validation-threshold search
Maximum epochs	200
Batch size	16; validation batch size 32
Mixed-precision	Enabled
Threshold selection	Validation search from 0.10 to 0.90; best threshold reported per run
Hardware	AutoDL server with 6 × NVIDIA RTX 4090D; one GPU per model process
Seed policy	Five random seeds for reported benchmark means; seed 3407 retained as the reference development run
Robustness check	Focused paired five-seed comparison between GeoSlide-XMamba and the strong 14-channel concat baseline

**Table 4:** (**a**) Main benchmark comparison on the Landslide4Sense validation split. Part a reports HD95 and computational cost, and part b reports precision, recall, region overlap, boundary metrics, model size, and training time. The downward arrow (↓) indicates that lower HD95 values are better. (**b**) Quantitative comparison on the Landslide4Sense validation split. P, precision; R, recall; and B-F1, Boundary-F1. Values are reported as five-seed means, while threshold selection was performed within each validation run.

(**a**)											
**Model**	**F1**		**IoU**			**B-F1**		**HD95 ↓**		**Params/FLOPs**	**Infer./GPU**
U-Net	0.627		0.452			0.421		4.90		7.77 M/12.4 G	5.3 ms/1.2 GB
Attention U-Net	0.645		0.471			0.425		4.59		16.07 M/24.1 G	8.1 ms/1.9 GB
GeoSlide-XMamba	0.673		0.507			0.466		3.45		57.60 M/52.3 G	13.8 ms/3.2 GB
(**b**)											
**Model**	**P**	**R**		**F1**	**IoU**		**Kappa**		**B-F1**	**Params (M)**	**Time (min)**
U-Net	0.661	0.596		0.627	0.452		0.619		0.421	7.77	79.03
ResU-Net	0.673	0.607		0.639	0.469		0.631		0.432	15.87	90.84
Attention U-Net	0.702	0.592		0.645	0.471		0.637		0.425	16.07	97.07
U-Net++	0.689	0.599		0.636	0.467		0.628		0.434	12.03	96.42
DeepLabV3+	0.691	0.602		0.635	0.462		0.627		0.431	12.06	93.49
ConvNeXt-UNet	0.686	0.604		0.631	0.459		0.623		0.426	7.13	90.55
PyrMamba-mini	0.648	0.553		0.596	0.429		0.588		0.315	12.31	92.95
GeoSlide-XMamba	0.729	0.626		0.673	0.507		0.666		0.466	57.60	276.40

Note: Results in Table 4 are unified with the five-seed benchmark statistics. Precision, recall, F1-score, IoU, kappa, and Boundary-F1 are averaged independently across seeds and rounded to three decimals; therefore, the displayed F1-score is not recomputed from the displayed mean precision and recall.

**Table 5:** Component sensitivity analysis for GeoSlide-XMamba on the validation split. The table reports five-seed mean values for the main architectural and loss function variants. The downward arrow (↓) indicates that lower HD95 values are better.

Variant	F1	IoU	Kappa	B-F1	HD95 ↓
Full GeoSlide-XMamba	0.673	0.507	0.666	0.466	3.45
w/o TC-SS2D	0.659	0.491	0.652	0.448	3.99
w/o SDF/HD95 loss	0.662	0.494	0.655	0.447	4.03
w/o hard-negative mining	0.666	0.501	0.659	0.456	3.72
w/o object-aware decoder	0.665	0.499	0.658	0.454	3.77
w/o deformable boundary	0.666	0.500	0.659	0.455	3.71

Note: HD95 is reported in pixels, and lower values indicate better boundary agreement.

**Table 6:** Input-modality analysis from the available runs. Values are harmonized with the benchmark statistics reported in Table 4.

Input Setting	Channels	F1	IoU	B-F1
RGB-only	3	0.548	0.381	0.346
Sentinel-2-only	12	0.597	0.424	0.387
14-channel concat strong	14	0.628	0.457	0.414
Full GeoSlide-XMamba	14	0.673	0.507	0.466

Note: Full GeoSlide-XMamba values are five-seed means consistent with the benchmark statistics reported in Table 4.

**Table 7:** (**a**) Five-seed robustness statistics and focused comparison between GeoSlide-XMamba and the strong 14-channel concatenation baseline. Part a reports mean, standard deviation, confidence intervals, HD95, and paired-test results; part b reports metric-wise robustness. (**b**) Metric-wise five-seed robustness comparison between GeoSlide-XMamba and the strong 14-channel concatenation baseline. Values are mean ± standard deviation.

(**a**)									
**Model**	**F1 Mean ± Std**	**F1 95% CI**	**IoU Mean ± Std**		**B-F1 Mean ± Std**		**HD95 Mean ± Std**		***p*-Value**
14-channel concat	0.628 ± 0.005	[0.622, 0.634]	0.457 ± 0.005		0.414 ± 0.009		4.87 ± 0.18		-
GeoSlide-XMamba	0.673 ± 0.003	[0.670, 0.676]	0.507 ± 0.002		0.466 ± 0.002		3.45 ± 0.13		0.002
(**b**)									
**Model**		**F1**		**IoU**		**Kappa**		**B-F1**	
14-channel concat strong		0.628 ± 0.005		0.457 ± 0.005		0.621 ± 0.005		0.414 ± 0.009	
GeoSlide-XMamba		0.673 ± 0.003		0.507 ± 0.002		0.666 ± 0.003		0.466 ± 0.002	

## Data Availability

The Landslide4Sense benchmark dataset is publicly available from the original dataset providers. The processed splits, trained weights, and code required to reproduce the experiments will be made available in a public repository upon acceptance, subject to data license restrictions. The Jinsha River regional data will be derived from public Sentinel-2 and DEM products. The source code for GeoSlide-XMamba will be made available upon reasonable request to the corresponding author.
